# Comprehensive analysis of MGLL as a novel diagnostic and prognostic biomarker for clear cell renal cell carcinoma

**DOI:** 10.3389/fonc.2025.1594540

**Published:** 2025-09-09

**Authors:** Chen Chen, Zhuojing Hu, Wei Zhao, Yunbo Ma, Qinghua Xia

**Affiliations:** ^1^ Department of Urology, Liaocheng People’s Hospital, Liaocheng, Shandong, China; ^2^ Medical Integration and Practice Center, Shandong University, Jinan, Shandong, China; ^3^ Liaocheng People’s Hospital Affiliated to Shandong First Medical University, Liaocheng, Shandong, China; ^4^ Department of Urology, Shandong Provincial Hospital Affiliated to Shandong First Medical University, Jinan, Shandong, China

**Keywords:** MGLL, clear cell renal cell carcinoma, immune infiltration, protein-protein interaction, m7G

## Abstract

**Background:**

Clear cell renal cell carcinoma (ccRCC) is a malignancy with significant morbidity and metabolic-related characteristics, necessitating the exploration of novel biomarkers and therapeutic targets. This study focuses on monoglyceride lipase (MGLL), an important molecule identified through RNA sequencing of lipid metabolism-related genes.

**Methods:**

We investigated MGLL expression and function in ccRCC by analyzing mRNA data, clinical information, and multiple databases. We used R packages to analyze gene co-expression, immune infiltration, and m7G methylation gene correlations. We constructed a protein-protein interaction (PPI) network and performed prognostic and diagnostic receiver operating characteristic (ROC) curve analyses to identify differentially expressed genes (DEGs). We further validated these genes by qRT-PCR and performed functional experiments by knocking down MGLL using lentiviral vectors.

**Results:**

Both qRT-PCR experiments and immunohistochemical data demonstrate that MGLL is upregulated in ccRCC tissues relative to normal tissues. The area under the curve (AUC) values from ROC analyses of three GEO validation datasets (GSE40435, GSE66270, and GSE213324) all exceeded 0.9. The expression of MGLL is associated with poor prognosis and correlates with gender and histological grade. Functional enrichment analysis showed that genes co-expressed with MGLL were mainly involved in proteasome-mediated protein degradation, macroautophagy, and the response to endoplasmic reticulum stress. MGLL expression is significantly positively correlated with the infiltration of neutrophils, Th17 cells, eosinophils, and dendritic cells. In contrast, it is significantly negatively correlated with cytotoxic T cells, NK CD56bright cells, and CD8 T cells. The PPI network and the correlation analysis between MGLL and m7G genes identified a total of 23 DEGs. Additionally, prognostic LASSO regression coefficients combined with ROC analysis reveal that ACLY, CALM3, NSUN2, NUDT16, NUDT4, and PKM have potential prognostic and diagnostic value. qRT-PCR experiments confirmed the expression of 13 genes from the prognostic LASSO model in ccRCC cell lines ACHN, A498, and 786-O, as well as in normal renal tubular epithelial cells HK-2. Inhibition of MGLL expression reduced ccRCC cell proliferation, colony formation, and migration.

**Conclusion:**

This investigation elucidates the diagnostic and prognostic significance of MGLL in ccRCC, while offering mechanistic insights into its biological functions and potential therapeutic implications.

## 1 Introduction

Renal cell carcinoma (RCC), with clear cell renal cell carcinoma (ccRCC) as its most prevalent histologic subtype, accounts for approximately 70-80% of all RCC cases and poses a substantial clinical challenge due to its increasing global incidence (1). Cancer pathogenesis is fundamentally governed by the dysregulation of oncogenes and tumor suppressor genes, often compounded by germline mutations. Over the past several decades, significant progress in elucidating the intricate molecular mechanisms underlying cancer biology has substantially contributed to the development of more effective therapeutic strategies (2). Early-stage ccRCC, particularly tumors confined to the kidney and measuring less than 4 cm, demonstrates a favorable prognosis, with a 5-year cancer-specific survival rate exceeding 94% following surgical interventions such as partial or radical nephrectomy or ablative therapies (3). In contrast, advanced or metastatic RCC is associated with a markedly poorer prognosis, exhibiting a 5-year survival rate of only 12% and a median overall survival (OS) ranging from 46 to 56 months. Notably, approximately 30% of patients present with metastatic disease at initial diagnosis, while an additional 30% develop metastases during follow-up (4). Although recent advancements in biological targeted therapies and immune checkpoint inhibitors have improved progression-free survival, significant challenges persist, including tumor resistance, metastatic progression, disease recurrence, suboptimal long-term response rates, and substantial financial burden (5, 6). In contrast to singularly targeting tumor cells, stromal cells, or immune cells, integrative multi-target therapeutic approaches circumvent limitations imposed by genomic instability, drug resistance, and the functional heterogeneity of immune cells. This underscores the critical role of the tumor microenvironment in therapeutic efficacy and provides a strategic framework for future research directions (7). The aggressive nature of ccRCC, coupled with the limitations of current therapeutic modalities, underscores the critical need for the development of novel treatment strategies and predictive biomarkers to improve clinical outcomes.

Emerging evidence has demonstrated that metabolic reprogramming in cancer cells, particularly characterized by dysregulated lipid metabolism, plays a pivotal role in the pathogenesis of ccRCC (8). Lipid accumulation, which serves as both a hallmark of metabolic dysregulation and a dynamic energy reservoir, significantly contributes to the formation of membrane structures that drive malignant phenotypes, including tumor cell proliferation and metastatic potential. This metabolic alteration facilitates oncogenic signaling pathways within cancer cells and modulates the tumor microenvironment (9). Building upon these findings, our research team previously developed a clinical prognostic risk model based on lipid storage-related genes, identifying AUP1 (Ancient Ubiquitous Protein 1), a lipid droplet regulator involved in VLDL assembly, as a key player in ccRCC progression through its induction of lipid accumulation (10). Subsequent RNA sequencing analysis revealed monoglyceride lipase (MGLL) as a critical downstream effector of AUP1. MGLL encodes a serine hydrolase belonging to the AB hydrolase superfamily, which catalyzes the hydrolysis of monoacylglycerides into free fatty acids (FAs) and glycerol, thereby playing a significant role in lipid metabolism and tumorigenic processes (11, 12).

Specific lipases are critically involved in the mobilization of FAs from lipid stores for cellular metabolism. Inhibition of lipolytic pathways reduces the availability of FAs, which are essential for cancer cell proliferation, while the FAs generated through lipolysis function as key precursors for signaling lipids (13). MGLL, a central enzyme in lipolysis, catalyzes the hydrolysis of monoacylglycerol into free FAs and glycerol. The liberated FAs are subsequently utilized in vital cellular processes, including membrane biosynthesis, lipid signaling, and energy storage. In ccRCC, inhibition of FA β-oxidation leads to the accumulation of FAs, which are preferentially directed toward membrane biogenesis and energy storage. This metabolic reprogramming ensures a continuous energy supply, thereby facilitating the rapid proliferation of tumor cells (14). Based on these observations, we propose that MGLL plays a significant role in mediating lipid accumulation in ccRCC cells. However, the precise mechanistic role of MGLL in ccRCC pathogenesis remains poorly characterized, warranting further investigation into its associated biological processes, molecular pathways, and expression patterns.

To elucidate the functional significance of MGLL in ccRCC, this study employed a multi-omics approach, integrating data from diverse public databases to conduct a systematic analysis of MGLL and its associated gene network. The expression profile of MGLL in ccRCC was assessed, alongside its correlation with clinicopathological features and diagnostic utility. Through differential expression and co-expression analyses, potential biological pathways and processes linked to MGLL were identified. Furthermore, the study explored the relationship between MGLL expression, tumor immune infiltration, and m7G methylation modifications. Candidate genes associated with MGLL were systematically screened and validated through experimental approaches. Collectively, these findings provide insights into the role of MGLL in ccRCC pathogenesis and highlight its potential as a biomarker for prognosis and therapeutic targeting in ccRCC.

## 2 Materials and methods

### 2.1 Data acquisition and analysis

The mRNA expression data and clinical information were downloaded from The Cancer Genome Atlas (TCGA) database (https://www.cancer.gov/ccg/research/genome-sequencing/tcga) and Genotype-Tissue Expression (GTEx) database (15). The TCGA database includes ccRCC tissues from 531 patients and adjacent normal tissues from 72 cases, while the GTEx database includes RNAseq data from 28 normal tissues (16). The extracted data is presented in Transcripts Per Million (TPM) format. The proteomics data and immunohistochemical staining slides are derived from the Clinical Proteomic Tumor Analysis Consortium (CPTAC) database (17) and The Human Protein Atlas (HPA) database (https://www.proteinatlas.org/) (18). The gene amplification and mutation status of MGLL was obtained from the cBioPortal (http://www.cbioportal.org/) for Cancer Genomics (19). The GEPIA database (http://gepia.cancer-pku.cn/) (20), the UALCAN database (https://ualcan.path.uab.edu/index.html) (21) and the TCGA database were utilized for Kaplan-Meier survival curve analysis. The three independent validation datasets, comprising comprehensive gene expression profiles of both ccRCC and matched normal control samples, were systematically retrieved from the Gene Expression Omnibus (GEO) database (https://www.ncbi.nlm.nih.gov/geo/). The GSE66270 dataset contains mRNA microarray expression analysis data from 28 pairs of matched malignant and non-malignant kidney tissue samples from 14 ccRCC patients without diagnosed metastasis (22). The GSE40435 dataset comprises comprehensive whole-genome expression profiles derived from 101 paired samples of ccRCC tumors and their corresponding adjacent non-tumor kidney tissues (23). Additionally, the GSE213324 dataset contains high-throughput sequencing data obtained from 21 RCC samples and 20 normal kidney tissue specimens (24). Detailed information regarding these datasets is presented in **Table 1**.

**Table 1:** Details of GEO data used in this study.

GEO datasets	ccRCC samples	Normal samples	Experiment type	Platforms
GSE66270	14	14	Array	GPL570
GSE40435	101	101	Array	GPL10558
GSE213324	21	20	High throughput sequencing	GPL24676

### 2.2 Genetic differences and gene set enrichment analysis

A threshold value of 50% was selected to categorize the cohort into high and low MGLL expression groups. The ‘ggplot2’ package of R software (version 4.2.1) is utilized to visualize the results of the differential analysis. The enrichment analysis of gene sets is performed using the ‘clusterProfiler’ package, including Gene Ontology (GO) analysis and Kyoto Encyclopaedia of Genes and Genomes (KEGG) pathway analysis. We performed Receiver Operating Characteristic (ROC) analysis on the data and visualized the results using the ‘pROC’ and ‘ggplot2’ packages. The ‘survival’ R package is used to conduct proportional hazards assumption tests and perform survival regression curve analysis, with results visualized using the ‘survminer’ and ‘ggplot2’ packages.

### 2.3 Correlation analysis and construction of protein-protein interaction network

Gene correlation analysis employs the Spearman method, and an absolute correlation coefficient greater than 0.3 indicates a correlation between the two. The STRING database (https://cn.string-db.org/) is used to construct a protein-protein interaction (PPI) network, and then visualize the data using Cytoscape software 3.9.1 (https://cytoscape.org/index.html) (25, 26). We further analyzed the PPI network and performed gene screening and mining using the Mcode plugin.

### 2.4 Immune infiltration analysis, extraction of m7G methylation modifications genes and prognostic analysis

The immune infiltration algorithm is based on the ssGSEA algorithm provided in the R package ‘GSVA’, which uses markers of 24 types of immune cells to calculate the immune infiltration status of the corresponding data. The stacked bar chart of immune infiltration is based on the CIBERSORT core algorithm. It utilizes markers of 22 types of immune cells provided by the CIBERSORTx website (https://cibersortx.stanford.edu/) to calculate the immune infiltration status of the data. The ‘ggplot2’ and ‘ggalluvial’ packages were used for computation and visualization. 29 m7G RNA modification-related regulator genes were extracted from the literature (27). We performed prognostic Least Absolute Shrinkage and Selection Operator (LASSO) coefficient selection by analyzing the cleaned data with the ‘glmnet’ package to obtain variable lambda values and visualize the results.

### 2.5 Cell culture, RNA extraction and real-time PCR

ACHN, A498, 786-O and HK-2 cells were purchased from the Chinese Academy of Sciences cell bank. All cells were cultured in the presence of penicillin/streptomycin at 37°C in air containing 5% CO_2_. Total RNA was isolated utilizing a commercial RNA extraction kit following the manufacturer’s protocol. Reverse transcription was carried out using the Evo M-MLV RT Premix. Quantitative PCR (qPCR) analysis was performed with the SYBR^®^ Green Premix Pro Taq HS qPCR kit. All RNA extraction, reverse transcription, and real-time fluorescence quantification reagents were procured from Accurate Biotechnology Co., Ltd. (Changsha, Hunan, China). PCR amplification was conducted using a thermal cycler (Bio-Rad Laboratories, USA), while fluorescence quantification was performed on the LightCycler 480II system (F. Hoffmann-La Roche Ltd, Switzerland). β-actin was used as an endogenous reference to normalize RNA expression. The relative expression of genes was calculated using the 2^−ΔΔCt^ method. The primer sequences can be found in the **Supplementary Table S1**.

### 2.6 Western blotting

Protein extraction was performed using RIPA lysis buffer (Beijing Solarbio Science & Technology Co., Ltd, China), followed by quantification of protein concentration through the Bicinchoninic Acid Assay (BCA) method, employing a gradient of standard samples to establish a calibration curve. Polyacrylamide gel electrophoresis (PAGE) was conducted using a gel preparation kit (Yzyme Biotech Co., Ltd, Shanghai, China), with protein samples (30 μg per lane) resolved on 6-15% sodium dodecyl sulfate-polyacrylamide gels and subsequently transferred to polyvinylidene fluoride (PVDF) membranes (MilliporeSigma, USA). Primary antibodies against MGLL (Catalog No.: ab124796, Abcam Inc., USA) and β-actin (Proteintech Group, Inc., China) were utilized, along with horseradish peroxidase-conjugated goat anti-rabbit/mouse secondary antibodies (Proteintech Group, Inc., China). Electrophoresis and transfer buffers were obtained from Yzyme Biotech Co., Ltd, Shanghai, China. Membranes were blocked with 5% non-fat milk, incubated overnight with primary antibodies, and subsequently probed with horseradish peroxidase-conjugated secondary antibodies. Protein bands were visualized using enhanced chemiluminescence (ECL) detection solution (MilliporeSigma, USA) in a gel imaging system.

### 2.7 Lentiviral transfection

Stable MGLL knockdown cell lines were established in ccRCC cell lines A498, 786-O and ACHN. To achieve stable transfection, si-MGLL was subcloned into the lentiviral vector (LV) GV493 (hU6-MCS-CBh-gcGFP-IRES-puromycin), along with the negative control virus (NC, CON313), both purchased from Shanghai Genechem Co., Ltd., China. Puromycin was used to screen for stable cell lines. Three RNA interference (RNAi) sequences were prepared for LV-si-MGLL: PSC91228-1 (CAACTCCGTCTTCCATGAAAT), PSC91229-1 (CCAGGACAAGACTCTCAAGAT), and PSC91230-1 (CCAATCCTGAATCTGCAACAA). The control insert sequence was TTCTCCGAACGTGTCACGT.

### 2.8 Cell proliferation, colony formation, and Transwell assay

The CCK-8 solution was prepared by diluting the CCK-8 reagent (Dojindo Molecular Technologies, Inc., Japan) in incomplete medium at a 10:1 volumetric ratio. Cells were seeded in a 96-well plate with five technical replicates per group and maintained in a standard cell culture incubator. Absorbance at 450 nm was quantified using a microplate reader at 0, 24, 48, and 72-hour time points. The experiment was performed in triplicate, and a standard curve was established for quantification. For the colony formation assay, cells were plated at a density of 600 cells per well in 6-well plates and cultured until microscopic examination revealed the formation of >50 single-cell colonies. Subsequently, cells were washed, fixed, and stained with 0.1% crystal violet (Beijing Solarbio Science & Technology Co., Ltd., China), followed by air-drying and photographic documentation. For the migration assay, cells were seeded in the upper chamber of a Transwell system (Corning Incorporated, NY, USA) containing 200 µL of serum-free medium, while the lower chamber was filled with complete medium supplemented with 20% fetal bovine serum as a chemoattractant. After 48 hours of incubation, cells in the upper chamber were fixed, stained with crystal violet for 30 minutes, and the membrane was mounted on glass slides, sealed, dried, and imaged for analysis.

### 2.9 Statistical analysis

All statistical analyses were performed using R software (version 4.2.1) alongside GraphPad Prism 9 software. Statistical significance for differences between groups was determined using the t-test. For comparisons among multiple groups, one-way analysis of variance (ANOVA) was used. A *p*-value of less than 0.05 was considered statistically significant. The following *p*-values were considered: *p < 0.05, **p < 0.01, and ***p < 0.001.

## 3 Results

### 3.1 The expression of MGLL in pan-cancer and ccRCC

**Figure 1:**
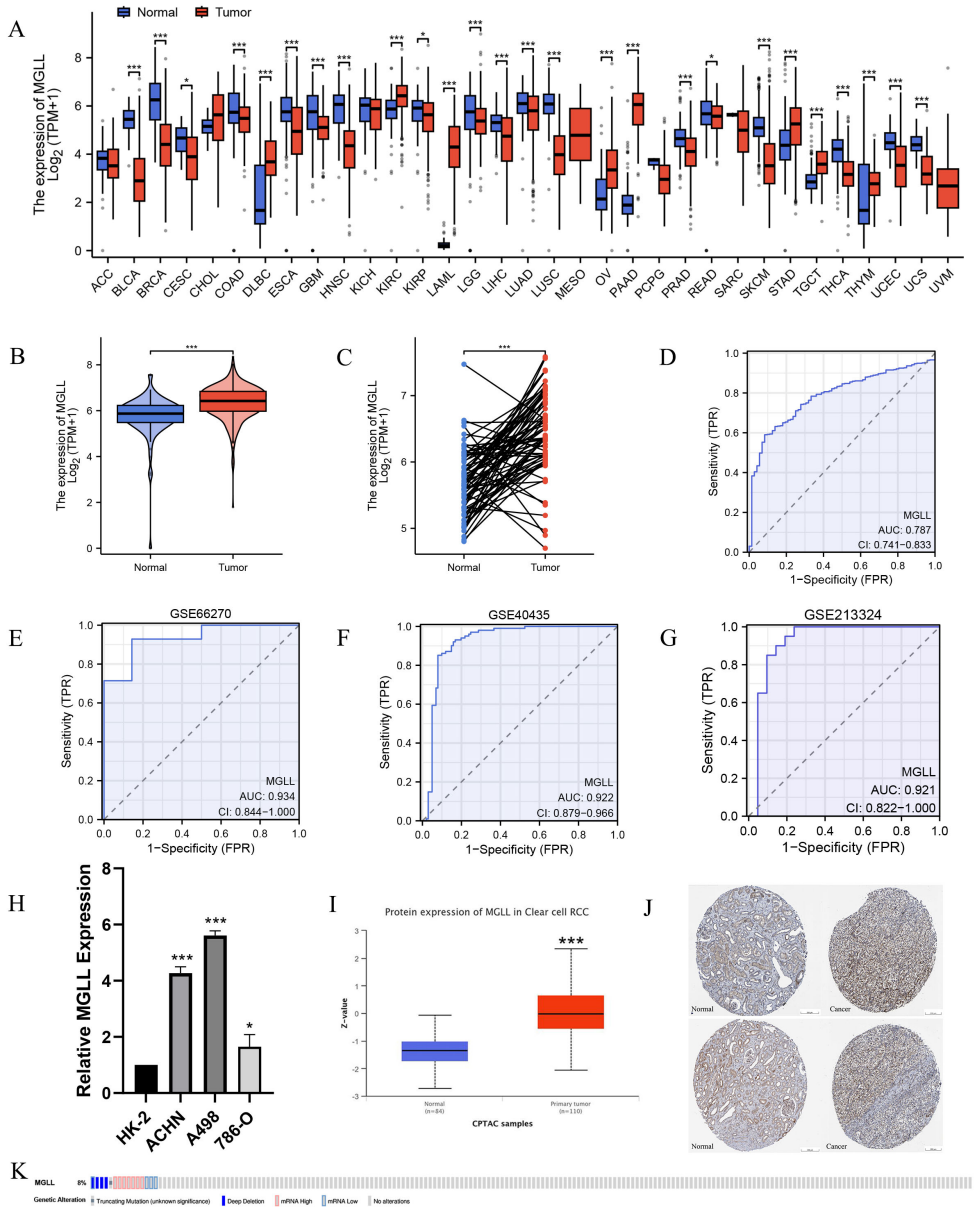
Expression levels of MGLL. **(A)** Expression levels of MGLL in pan-cancer and corresponding normal tissues from TCGA and GTEx databases. **(B)** Differential expression of MGLL between ccRCC (n = 531) and normal tissues (n = 100, 72 cases of adjacent tissues from TCGA and 28 cases of normal tissues from GTEx). **(C)** The expression of MGLL in ccRCC and normal paired tissues (n = 72). **(D)** ROC curve for the diagnosis of MGLL (AUC = 0.787). **(E)** Diagnostic ROC curve of MGLL in the GSE66270 dataset (AUC = 0.934). **(F)** Diagnostic ROC curve of MGLL in the GSE40435 dataset (AUC = 0.922). **(G)** Diagnostic ROC curve of MGLL in the GSE213324 dataset (AUC = 0.921). **(H)** qRT-PCR assay of mRNA expression levels of MGLL in HK-2 and ccRCC cell lines (ACHN, A498 and 786-O). **(I)** The protein expression levels of MGLL in ccRCC (n = 110) and normal tissues (n = 84) in the CPTAC database. **(J)** Immunohistochemical staining of MGLL in ccRCC and normal tissues (scale bar, 200 μm). **(K)** cBioPortal OncoPrint graph showing the distribution of MGLL genomic changes in ccRCC patients. *p < 0.05; ***p < 0.001.

To explore the significance of MGLL in tumor tissues, we first analyzed its expression in pan-cancer tissues. The results indicated statistically significant differences in MGLL expression in 26 of the 33 tumor types compared to normal tissues (**Figure 1A**). MGLL was significantly upregulated in diffuse large B cell lymphoma (DLBC), acute myeloid leukemia (LAML), ovarian serous cystadenocarcinoma (OV), pancreatic adenocarcinoma (PAAD), and stomach adenocarcinoma (STAD), while it was downregulated in breast invasive carcinoma (BRCA), head and neck squamous cell carcinoma (HNSC), lung squamous cell carcinoma (LUSC), bladder urothelial carcinoma (BLCA), and skin cutaneous melanoma (SKCM). RNA-seq data from the TCGA and GTEx databases show that MGLL mRNA levels are significantly elevated in ccRCC compared to normal tissues (**Figures 1B, C**). We used ROC curve analysis to evaluate the effectiveness of MGLL in distinguishing between tumor and non-tumor tissues. The MGLL gene demonstrated an area under the curve (AUC) value of 0.787 in initial analyses (**Figure 1D**). Subsequent validation across three independent GEO datasets revealed good diagnostic performance, with ROC curve analyses yielding AUC values of 0.934 (GSE66270) (**Figure 1E**), 0.922 (GSE40435) (**Figure 1F**), and 0.921 (GSE213324) (**Figure 1G**). These consistently high AUC values (> 0.9) across multiple datasets suggest that MGLL expression exhibits significant potential as a biomarker for distinguishing tumor from non-tumor tissues. Further confirmation through qRT-PCR experiments showed that compared to normal renal tubular epithelial cells HK-2, the mRNA level of MGLL was significantly elevated in ccRCC cell lines ACHN, A498 and 786-O (**Figure 1H**). The CPTAC protein database indicates that the protein level of MGLL is significantly higher in ccRCC tissues compared to normal tissues (**Figure 1I**). Immunohistochemical staining in the HPA database indicates that the protein level of MGLL in ccRCC tissues is higher than that in normal renal tissues (**Figure 1J**). In addition, to explore the mutation level of MGLL in ccRCC, we analyzed its genome and copy number. Analysis of the MGLL gene’s OncoPrint profile in ccRCC patients using the cBioPortal database revealed that truncating, deep deletion, and profiled mutations in MGLL were present in less than 8% of cases (**Figure 1K**).

### 3.2 The correlation between MGLL expression levels and clinicopathological characteristics in ccRCC patients

The baseline data of ccRCC patients from the TCGA database were statistically analyzed (**Table 2**). The data was divided into two groups: 270 ccRCC patients with low MGLL expression and 271 with high MGLL expression. Significant differences were observed between the two groups in histologic grade (*p* = 0.007) and gender (*p* < 0.001). No significant differences were found in TNM staging and age. Logistic regression analysis was conducted to examine the correlation between MGLL expression and the clinical pathological features of ccRCC (**Table 3**). Similar to the baseline statistics, the logistic regression analysis showed differences between the two groups in terms of the histologic grade (*p* = 0.043) and gender (*p* < 0.001).

**Table 2:** Baseline data table of MGLL expression and clinicopathologic characteristics in ccRCC.

Characteristics	Low expression of MGLL	High expression of MGLL	*P* value
n	270	271	
Age, n (%)			0.966
<= 60	134 (24.8%)	135 (25%)	
> 60	136 (25.1%)	136 (25.1%)	
Gender, n (%)			***< 0.001***
Female	60 (11.1%)	127 (23.5%)	
Male	210 (38.8%)	144 (26.6%)	
Pathologic T stage, n (%)			0.465
T1	135 (25%)	144 (26.6%)	
T2&T3&T4	135 (25%)	127 (23.5%)	
Pathologic N stage, n (%)			0.873
N0	116 (45%)	126 (48.8%)	
N1	8 (3.1%)	8 (3.1%)	
Pathologic M stage, n (%)			0.391
M0	211 (41.5%)	218 (42.9%)	
M1	43 (8.5%)	36 (7.1%)	
Pathologic stage, n (%)			0.301
Stage I	130 (24.2%)	143 (26.6%)	
Stage II&Stage III&Stage IV	138 (25.7%)	127 (23.6%)	
Histologic grade, n (%)			***0.007***
G1	2 (0.4%)	12 (2.3%)	
G2&G3&G4	263 (49.3%)	256 (48%)	

Bold p-values denote statistically significant differences (p < 0.05).

**Table 3:** Logistic regression analysis.

Characteristics	Total (N)	OR (95% CI)	*P* value
Age (> 60 vs. <= 60)	532	1.062 (0.756 – 1.492)	0.729
Gender (Male vs. Female)	532	0.331 (0.228 – 0.480)	**< 0.001**
Pathologic T stage (T3&T4 vs. T1&T2)	532	0.922 (0.647 – 1.313)	0.651
Pathologic N stage (N1 vs. N0)	256	0.935 (0.340 – 2.574)	0.897
Pathologic M stage (M1 vs. M0)	500	0.810 (0.500 – 1.312)	0.391
Histologic grade (G3&G4 vs. G1&G2)	524	0.701 (0.496 – 0.990)	**0.043**

Bold p-values denote statistically significant differences (p < 0.05).

**Figure 2:**
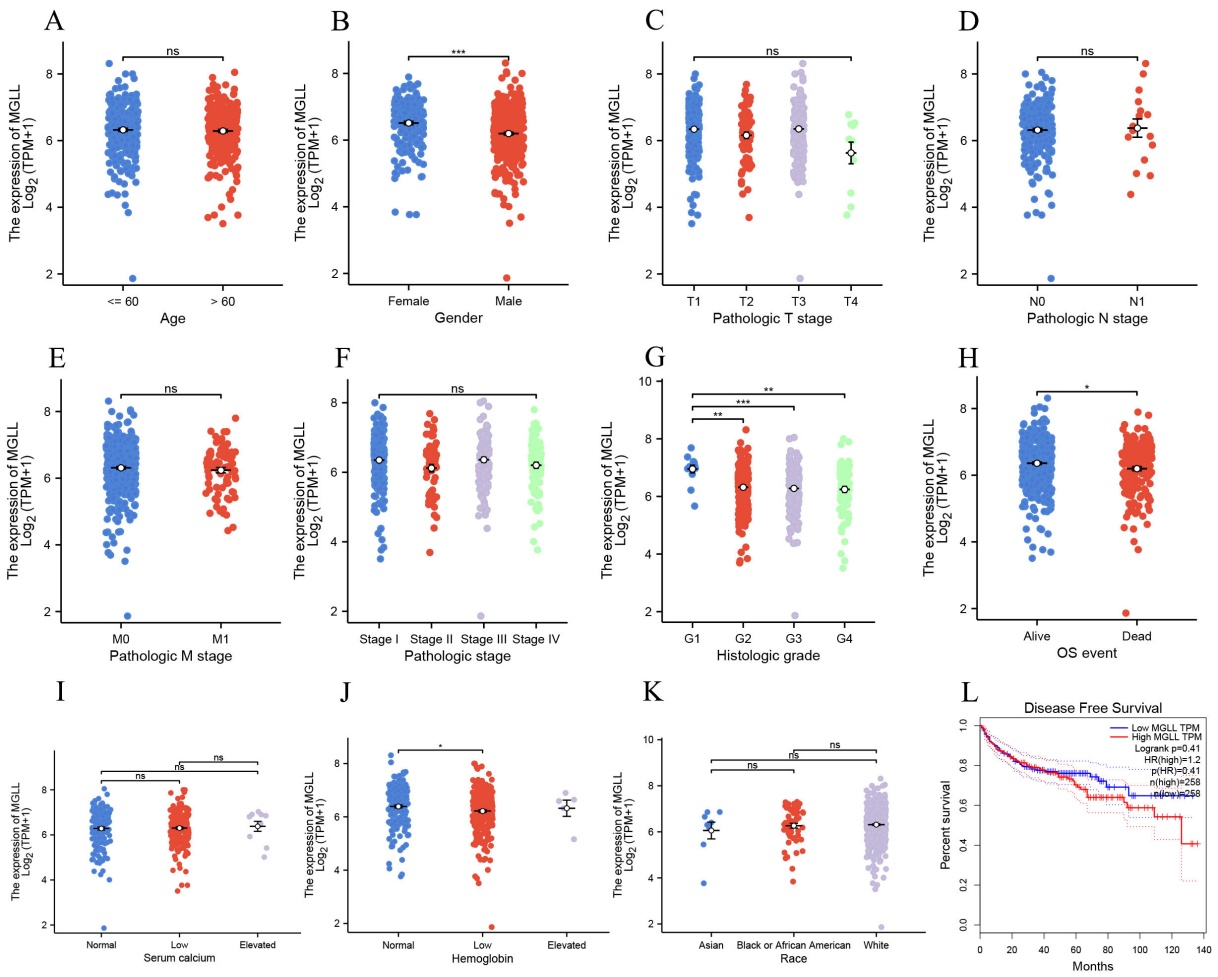
The expression levels in ccRCC patients with different clinical features of MGLL in TCGA database. **(A)** age, **(B)** gender, **(C)** pathologic T stage, **(D)** pathologic N stage, **(E)** pathologic M stage, **(F)** pathologic stage, **(G)** histologic grade, **(H)** OS event, **(I)** serum calcium, **(J)** hemoglobin, **(K)** race. **(L)** Analysis of disease progression-free survival curves for ccRCC patients based on the GEPIA database. *p < 0.05; **p < 0.01; ***p < 0.001; ns, no significance.

After confirming the increased expression of MGLL in ccRCC tissues, an exploration was conducted on the relationship between the expression of MGLL and 11 clinical variable characteristics within the ccRCC patient cohort. The variables included age, gender, T stage, N stage, M stage, pathologic stage, histologic grade, OS event, serum calcium, hemoglobin levels, and race (**Figure 2**). The results indicated that MGLL expression correlated with gender (**Figure 2B**), histologic grade (**Figure 2G**), OS event (**Figure 2H**), and hemoglobin (**Figure 2J**). Further analysis through disease-free survival curves indicated that high levels of MGLL expression were associated with decreased survival rates (**Figure 2L**), suggesting that the elevation of MGLL is related to clinical prognosis.

### 3.3 Functional clustering and PPI network analysis

**Figure 3:**
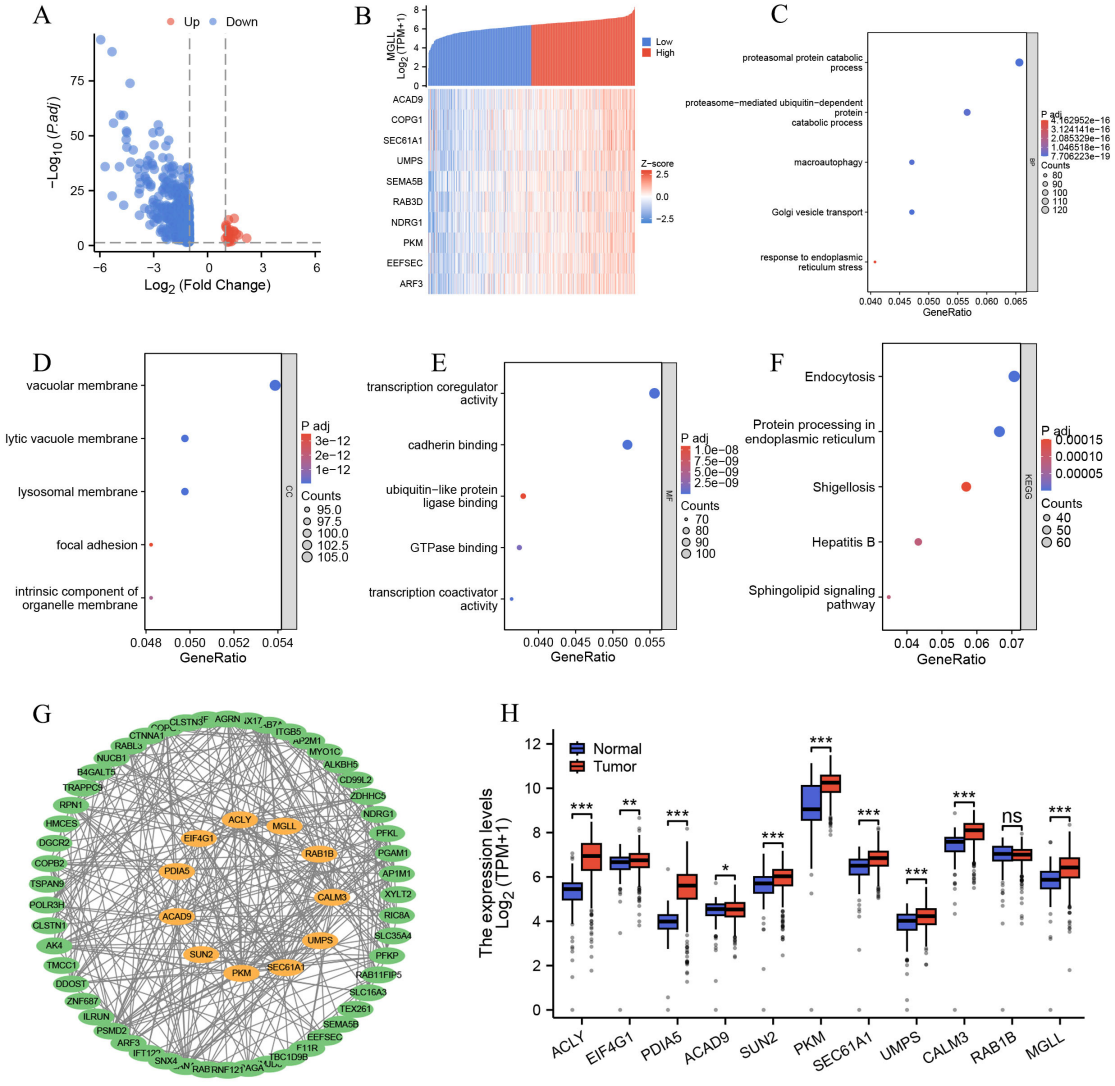
Functional enrichment and PPI network analysis of MGLL-related genes. **(A)** Volcano plot of related genes after MGLL differential analysis (|logFC| > 1, *p.adj* < 0.05) (n = 751). **(B)** Heatmap for co-expression showing the top 10 genes associated with MGLL. **(C-F)** Functional enrichment analysis of the biological processes, cellular components, molecular functions and KEGG pathways of MGLL-related genes (Spearman rank correlation coefficient > 0.4, *p.adj* < 0.05, n = 2008). **(G)** Analysis of PPI network and MCODE module of MGLL-related genes (Spearman correlation > 0.55, *p.adj* < 0.05, n = 65). The genes marked in orange in the middle are those analyzed by the MCODE module. **(H)** The expression of 11 genes analyzed by MCODE module in ccRCC and normal tissues. *p < 0.05; **p < 0.01; ***p < 0.001; ns, no significance.

We categorized ccRCC patients into two groups based on MGLL expression levels: high and low. We then performed a differential analysis. The screening criteria were established as follows: protein-coding genes, |logFC| > 1, and *p.adj* < 0.05. We screened a total of 751 differentially expressed genes, which included 22 upregulated genes and 729 downregulated genes (**Figure 3A**). Detailed information on all genes can be found in **Supplementary Table S2**. Next, we analyzed the co-expressed genes related to MGLL expression in the TCGA ccRCC dataset. The screening criteria were set as follows: |Spearman correlation| >0.4 and *p.adj* < 0.05. A total of 2008 genes were obtained, including 1 negatively correlated gene and the rest being positively correlated genes. For specific information on genes, please refer to **Supplementary Table S3**. The heatmap of the 10 genes with Spearman correlation > 0.6 co-expressed with MGLL is shown in **Figure 3B**. After conducting GO clustering analysis on the 2008 co-expressed genes of MGLL, the top five major biological processes involved are: proteasomal protein catabolic process, proteasome-mediated ubiquitin-dependent protein catabolic process, macroautophagy, Golgi vesicle transport, and response to endoplasmic reticulum stress (ERs) (**Figure 3C**). The key cellular components include the vacuolar membrane, lytic vacuole membrane, lysosomal membrane, focal adhesion, and intrinsic component of the organelle membrane (**Figure 3D**). The involved molecular functions mainly include: transcription coregulator activity, cadherin binding, ubiquitin-like protein ligase binding, GTPase binding, and transcription coactivator activity (**Figure 3E**). The main KEGG pathways involved include: Endocytosis, Protein processing in ER, Shigellosis, Hepatitis B, and Sphingolipid signaling pathway (**Figure 3F**). The detailed results of the GO analysis and KEGG pathway analysis can be found in **Supplementary Table S4**.

To further explore the genes that are strongly co-expressed with MGLL and their functional roles, we constructed a PPI network using the STRING database for 65 genes with a Spearman correlation greater than 0.55 (**Supplementary Table S3**), and visualized it using Cytoscape software (**Figure 3G**). We conducted module analysis on the network using the MCODE plugin to identify highly interconnected clusters of nodes. These clusters were scored based on node connectivity density and the number of nodes, facilitating the discovery of functionally related modules. The results show a module composed of 11 genes: ACLY, EIF4G1, PDIA5, ACAD9, SUN2, PKM, SEC61A1, UMPS, CALM3, RAB1B, and MGLL (**Figure 3G**). Then, the expression of these genes in ccRCC and normal tissues was analyzed through the TCGA and GTEx databases, revealing that, except for RAB1B, the other 10 genes exhibited significant differential expression (*p* < 0.05) (**Figure 3H**). This indicates that genes strongly associated with MGLL play important roles in ccRCC.

### 3.4 Correlation of MGLL expression with tumor immune infiltration

**Figure 4:**
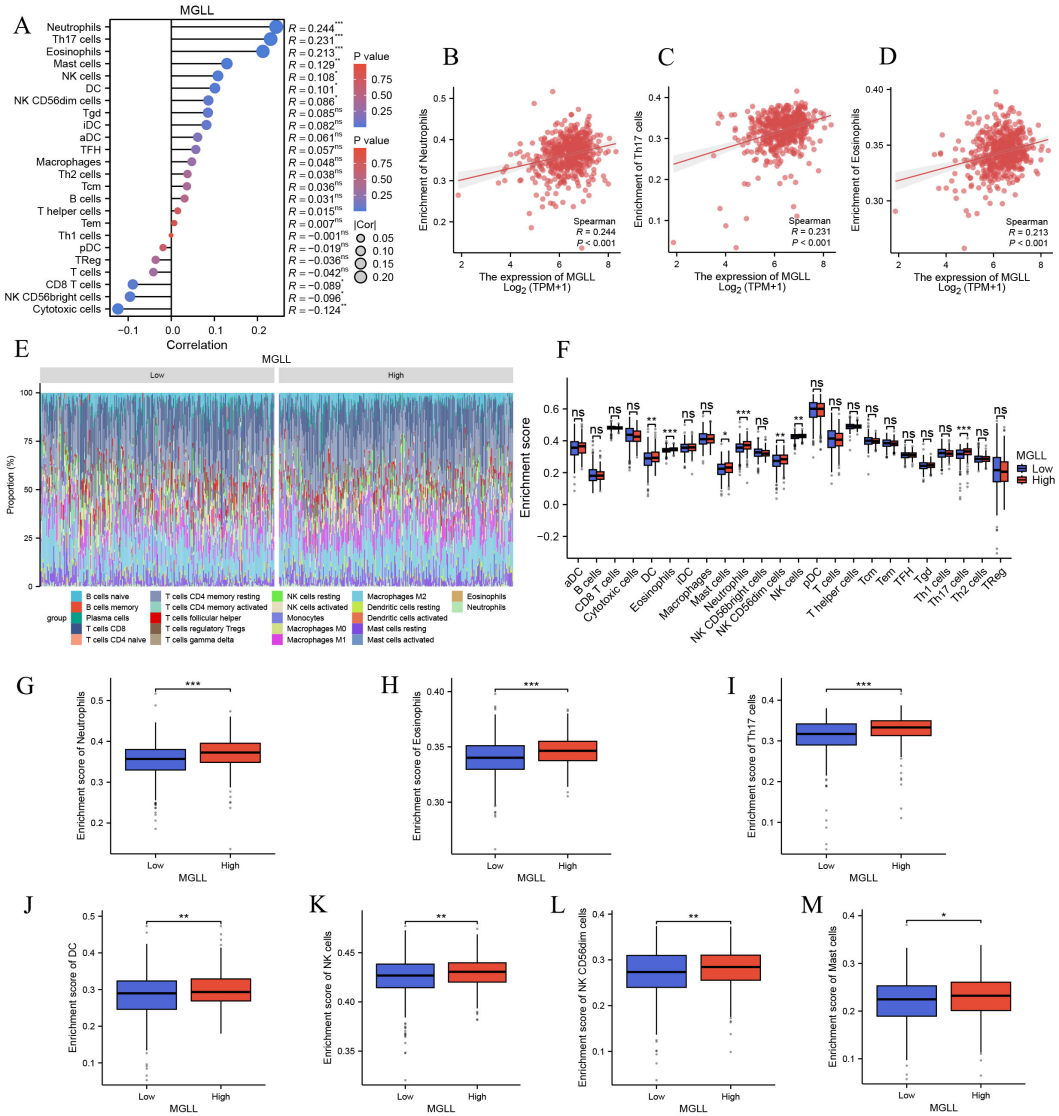
Correlation analysis of MGLL expression and immune infiltration in ccRCC. **(A)** The correlation between MGLL and immune infiltrating cells in ccRCC. **(B-D)** Scatter plot of the correlation between the expression of MGLL and the infiltration level of neutrophils, Th17 cells and eosinophils respectively. **(E)** Stacked bar chart of immune-infiltrating cells with high and low expression of MGLL in ccRCC. **(F)** Differential distribution of immune cells in patients with high and low MGLL expression. **(G-M)** Histograms showing the differences in the infiltration levels of neutrophils, eosinophils, Th17 cells, DC, NK cells, NK CD56dim cells and mast cells between high-expression and low-expression MGLL groups. *p < 0.05; **p < 0.01; ***p < 0.001; ns, no significance.

To explore the correlation between MGLL expression levels and tumor immune response, TCGA database was used to investigate immune infiltration in ccRCC with different MGLL expression levels. Analysis of 24 immune cell types revealed that MGLL expression in ccRCC patients is positively correlated with several immune cells, including Neutrophils, Th17 cells, Eosinophils, Mast cells, NK cells, dendritic cells (DCs), and NK CD56dim cells. Conversely, MGLL expression is negatively correlated with Cytotoxic cells, NK CD56bright cells, and CD8 T cells (*p* < 0.05) (**Figure 4A**). Further studies showed that MGLL expression was significantly positively correlated with Neutrophils (*p* < 0.001), Th17 cells (*p* < 0.001) and Eosinophils (*p* < 0.001) infiltration levels (**Figures 4B-D**). Based on the median expression level of MGLL, ccRCC patients were divided into high and low expression groups, and then the distribution of 22 immune cell subtypes (including 7 types of T cells, naive and memory B cells, plasma cells, NK cells, and bone marrow subpopulations) in the two groups was analyzed (**Figure 4E**). We also observed significant differences in the infiltration levels of Neutrophils, Th17 cells, Eosinophils, DC, NK CD56dim cells, NK cells, and Mast cells between the high and low MGLL expression groups (*p* < 0.05) (**Figures 4F-M**).

### 3.5 Association of MGLL expression levels with m7G methylation modifications in ccRCC

**Figure 5:**
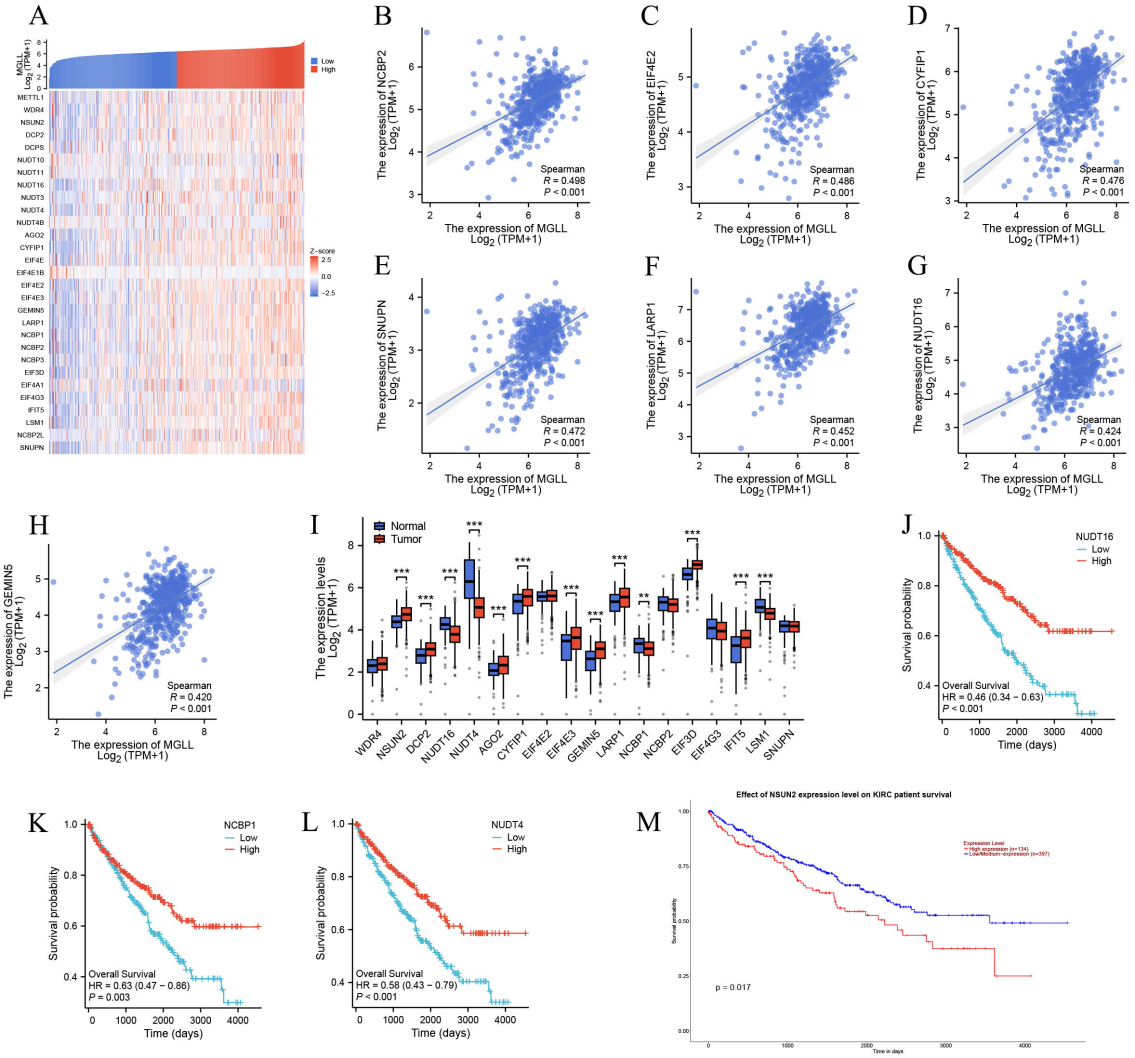
Association of MGLL expression with m7G methylation-related genes in ccRCC. **(A)** TCGA ccRCC cohort analyzed the association between MGLL and the expression of 29 m7G-related genes. **(B-H)** The scatter plot showing the correlation between MGLL and the expression of m7G-related genes including NCBP2, EIF4E2, CYFIP1, SNUPN, LARP1, NUDT16 and GEMIN5 in ccRCC. **(I)** The expression levels of 18 m7G-related genes (with a correlation coefficient r > 0.3) associated with MGLL in ccRCC (n = 537) and normal tissues (n = 72). **(J-M)** The survival curve analysis of NUDT16, NCBP1, NUDT4 and NSUN2 in ccRCC. **p < 0.01; ***p < 0.001.

The functional enrichment of genes co-expressed with MGLL includes biological processes such as mRNA processing, RNA catabolism process, RNA localization, RNA splicing, and RNA transport (*p.adj* < 0.001), suggesting that MGLL expression may be linked to RNA methylation, which plays a vital role in splicing, export, processing, translation, and degradation. m7G methylation, one of the most prevalent RNA modifications, has recently garnered significant attention as an emerging area of research (28–30). However, its specific molecular role in ccRCC is still lacking in-depth research. We aim to explore the differential expression and functional role of m7G methylated genes associated with MGLL in ccRCC. We first evaluated the co-expression heatmap of MGLL and 29 m7G modification genes, finding statistically significant correlations for 25 of these genes (Spearman *p* < 0.001) (**Figure 5A**) (**Supplementary Table S5**). The expression of MGLL was significantly positively correlated with NCBP2 (r = 0.498, *p* < 0.001) (**Figure 5B**), EIF4E2 (r = 0.486, *p* < 0.001) (**Figure 5C**), CYFIP1 (r = 0.476, *p* < 0.001) (**Figure 5D**), SNUPN (r = 0.472, *p* < 0.001) (**Figure 5E**), LARP1 (r = 0.452, *p* < 0.001) (**Figure 5F**), NUDT16 (r = 0.424, *p* < 0.001) (**Figure 5G**), and GEMIN5 (r = 0.42, *p* < 0.001) (**Figure 5H**). The scatter plot of genes with a correlation coefficient r > 0.3 related to MGLL expression can be found in **Supplementary Figure S1**. Subsequently, the expression of 18 genes with a correlation of r > 0.3 was analyzed in ccRCC and normal tissues using the TCGA and GTEx databases, revealing that 13 genes exhibited significant differential expression (*p* < 0.01) (**Figure 5I**). We also observed the prognostic associations of NUDT16, NCBP1, NUDT4, and NSUN2 in ccRCC (**Figures 5J-M**), suggesting a potential regulatory relationship with MGLL, which may affect patient prognosis.

### 3.6 Screening and diagnostic value of MGLL related genes, validation of GEO database

**Figure 6:**
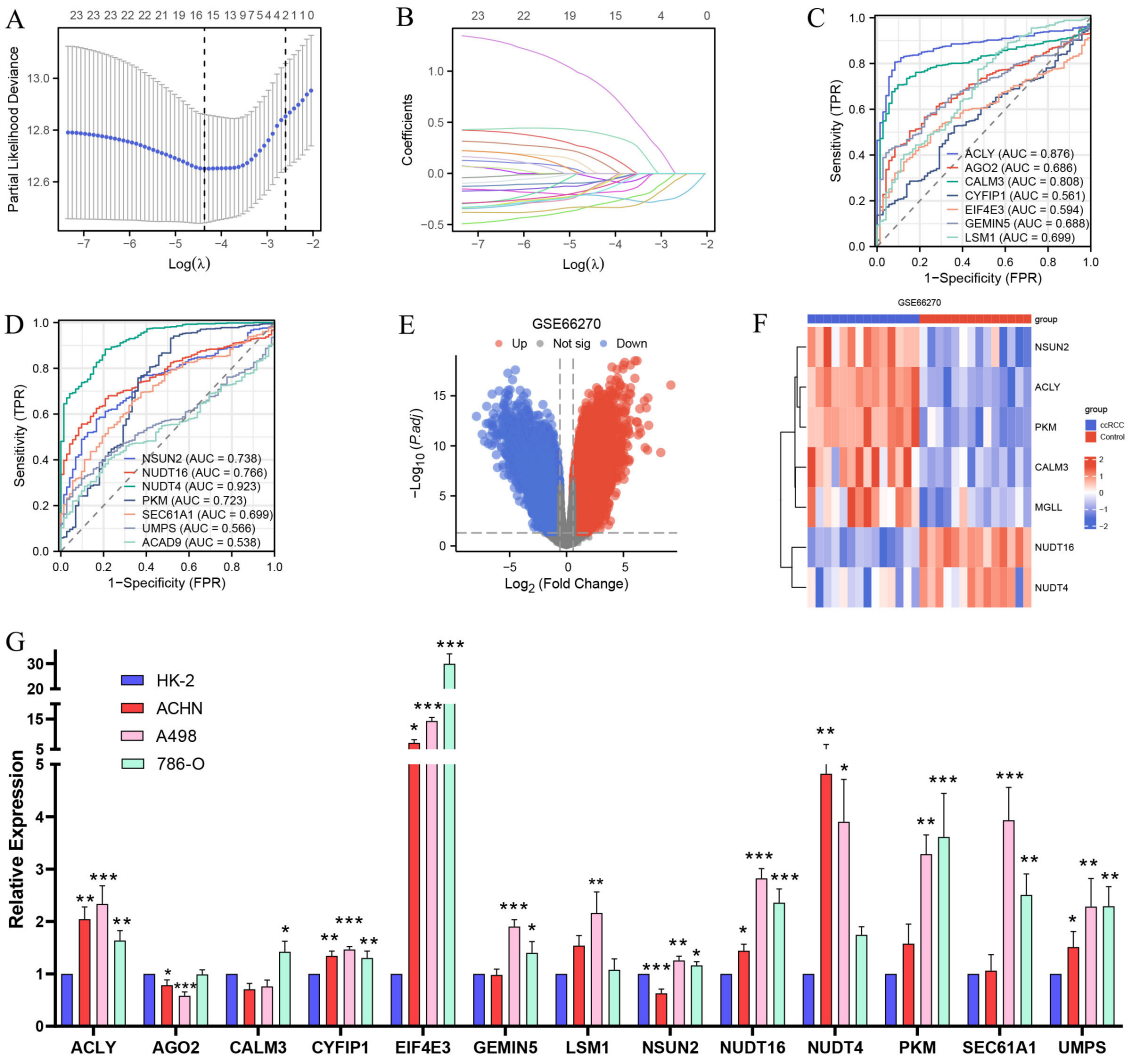
Validation of the GEO database, screening of prognostic and diagnostic biomarkers for MGLL-related genes, and qPCR experimental detection. **(A, B)** Prognostic LASSO coefficient screening and variable trajectory analysis for 23 MGLL-related genes (including MCODE analysis of PPI network and m7G-related genes). **(C, D)** ROC curve for diagnosis of MGLL-related genes (14 genes selected by prognostic LASSO coefficient) in ccRCC. **(E)** Volcano plots of differentially expressed genes in the dataset of ccRCC and normal tissues (GSE66270) (|logFC| > 0.58, *p.adj* < 0.05). **(F)** Heatmap of the expression of 7 prognostic and diagnostic-related genes shown in the GSE66270 dataset. **(G)** qRT-PCR experiment assay for the expression of the genes screened by the LASSO regression coefficient for prognosis in normal renal tubular epithelial cells HK-2 and ccRCC cell lines ACHN, A498 and 786-O. *p < 0.05; **p < 0.01; ***p < 0.001.

Based on the construction of the PPI network and the identification of m7G methylation genes related to MGLL, as well as the analysis using the TCGA and GTEx databases, a total of 23 genes including MGLL were found to have differential expressions in ccRCC and normal tissues (*p* < 0.05) (**Figures 3H** and **5I**). We further conducted LASSO coefficient screening for the 23 genes in combination with clinical prognosis using the ‘glmnet’ R package. Through the LASSO regression analysis, a risk signature model based on minimum criteria was constructed with 15 optimal and most powerful prognostic markers, which are: ACAD9, ACLY, AGO2, CALM3, CYFIP1, EIF4E3, GEMIN5, LSM1, MGLL, NSUN2, NUDT16, NUDT4, PKM, SEC61A1, and UMPS (**Figures 6A, B**). We further observed the performance of these genes in the TCGA database through diagnostic ROC curves to assess the diagnostic value of the genes in ccRCC. The results indicate that the AUC values for ACLY, CALM3, NSUN2, NUDT16, NUDT4, and PKM exceed 0.7, demonstrating high accuracy and sensitivity (**Figures 6C, D**). To verify the validity of their expression in ccRCC tissues, we switched databases and conducted a repeated validation using the dataset GSE66270 from the GEO database. After data cleaning, organization, and analysis, the GSE66270 dataset contained 9,465 upregulated genes and 6,931 downregulated genes (**Figure 6E**). The heatmap showed that compared to normal tissues, ACLY, CALM3, NSUN2, PKM, and MGLL were significantly upregulated, while NUDT16 and NUDT4 were significantly downregulated in ccRCC tissues (**Figure 6F**), confirming the results of the previous analysis.

### 3.7 Verify the expression of MGLL-related prognostic genes in various ccRCC cell lines through qRT-PCR experiments

We conducted qRT-PCR experiments on the genes selected based on the prognostic LASSO coefficients. The results showed that, compared to normal renal tubular epithelial cells HK-2, the expression levels of ACLY, CYFIP1, EIF4E3, and UMPS were significantly upregulated in ccRCC cell lines ACHN, A498, and 786-O. GEMIN5, NSUN2, PKM, and SEC61A1 were significantly upregulated in A498 and 786-O, and CALM3 was also upregulated in 786-O (**Figure 6G**), which is consistent with our previous analysis. In contrast, the expression of NUDT4 and NUDT16 in ccRCC cell lines was found to be increased, which contradicts our consideration of them as downregulated genes. Since both genes are involved in m7G methylation and belong to the nudix hydrolase family, the mRNA of RNA methyltransferases may be tagged by themselves or other enzymes, which could promote degradation or inhibit translation (31). Additionally, several alternative splicing transcriptional variants of NUDT4 have been described (32), and its selective splicing or translation variants may also lead to reduced protein levels. In summary, the qRT-PCR experiments validated the results of our previous analysis.

### 3.8 Prognostic model risk characteristics and evaluation

**Figure 7:**
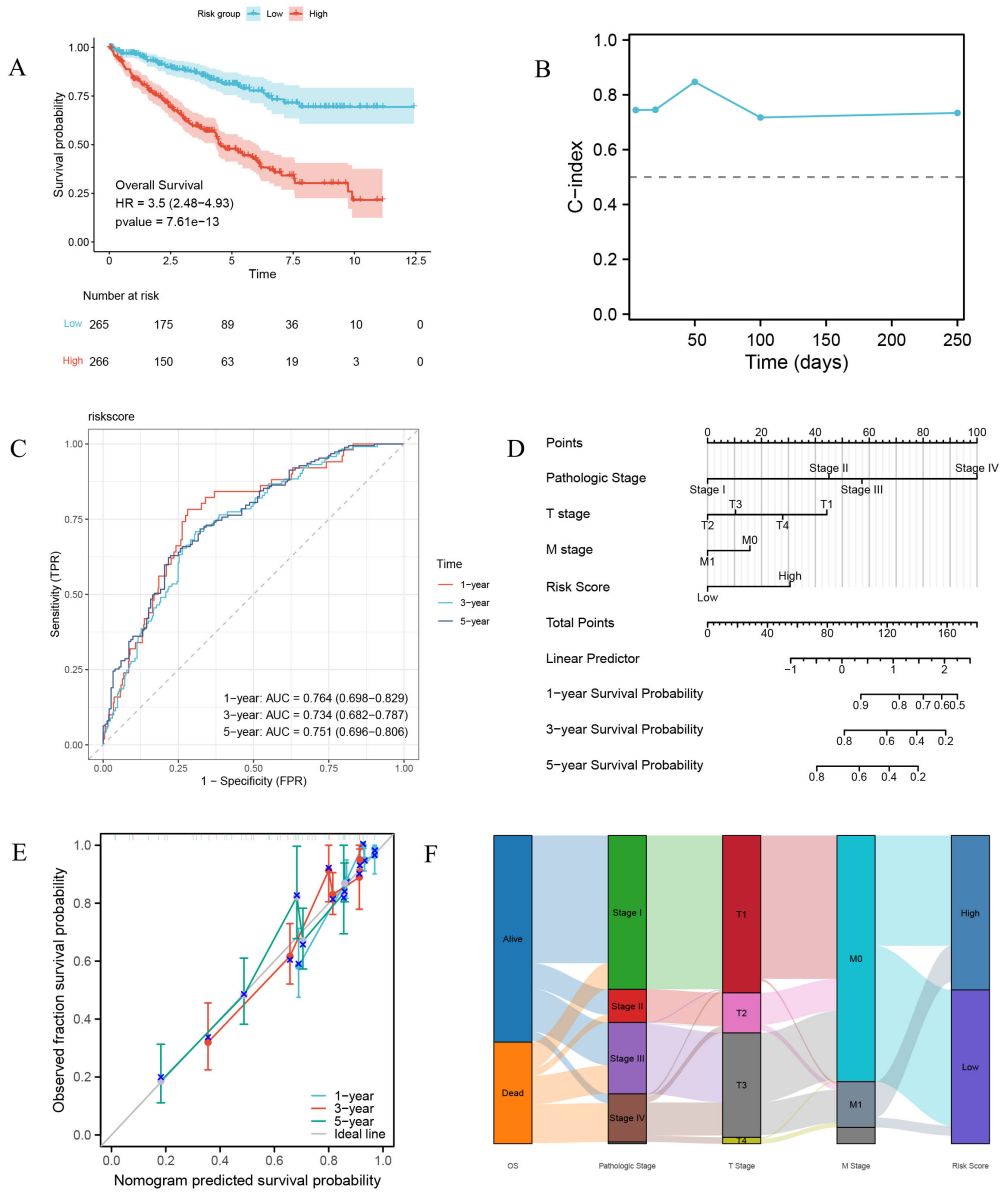
Prognostic risk characteristics and evaluation. **(A)** Survival curves of high-risk and low-risk group patients. **(B)** Time-dependent C-index plot of the risk model. **(C)** Time-dependent ROC curve of the risk model. **(D)** Prognostic nomogram combining risk scores and major clinical pathological variables. **(E)** Prognostic calibration curve. **(F)** Sankey diagram of clinical pathological features.

The LASSO-derived prognostic risk score stratified patients into distinct high-risk and low-risk cohorts, with Kaplan-Meier survival analysis demonstrating significantly poorer outcomes in the high-risk group (**Figure 7A**). Subsequent integration of the risk score with key clinical parameters, including Pathologic Stage and TM classification, through univariate and multivariate Cox regression analyses revealed the risk score as an independent prognostic indicator (*p* < 0.001) (**Table 4**), underscoring its clinical utility. Based on the multivariate regression analysis, the C-index at different time points showed that the model has relatively stable predictive performance (**Figure 7B**). Time-dependent ROC curve analysis yielded AUC values exceeding 0.7 at 1-, 3-, and 5-year intervals, indicating superior sensitivity and specificity (**Figure 7C**). The prognostic nomogram, incorporating both clinical variables and risk score, demonstrated accurate prediction of patient survival probabilities at 1, 3, and 5 years (**Figure 7D**), with calibration curves confirming excellent model fit (**Figure 7E**). The Sankey diagram illustrates the flow and proportional relationships of the multi-level association data among relevant clinical variables, survival, and risk scores (**Figure 7F**).

**Table 4:** Univariate and multivariate Cox analyses incorporating risk score from prognostic model and clinicopathological variables.

Characteristics	Total(N)	Univariate analysis		Multivariate analysis	
		Hazard ratio (95% CI)	*P* value	Hazard ratio (95% CI)	*P* value
Pathologic Stage	528				
Stage I	265	Reference		Reference	
Stage III	123	2.602 (1.736 - 3.900)	**< 0.001**	5.327 (2.002 - 14.172)	**< 0.001**
Stage II	57	1.188 (0.640 - 2.202)	0.585	3.725 (0.992 - 13.988)	0.051
Stage IV	83	6.503 (4.453 - 9.496)	**< 0.001**	18.530 (2.962 - 115.939)	**0.002**
T stage	531				
T1	271	Reference		Reference	
T3	180	3.261 (2.314 - 4.597)	**< 0.001**	0.372 (0.149 - 0.929)	**0.034**
T2	69	1.489 (0.894 - 2.481)	0.126	0.275 (0.085 - 0.883)	**0.030**
T4	11	10.434 (5.274 - 20.643)	**< 0.001**	0.620 (0.199 - 1.937)	0.411
M stage	503				
M0	424	Reference		Reference	
M1	79	4.361 (3.197 - 5.949)	**< 0.001**	0.633 (0.132 - 3.040)	0.568
Risk Score	531				
High	266	Reference		Reference	
Low	265	0.286 (0.203 - 0.403)	**< 0.001**	0.409 (0.284 - 0.589)	**< 0.001**

Bold p-values denote statistically significant differences (p < 0.05).

### 3.9 Suppression of MGLL expression attenuated the proliferative capacity, clonogenic potential, and migratory activity of ccRCC cells.

**Figure 8:**
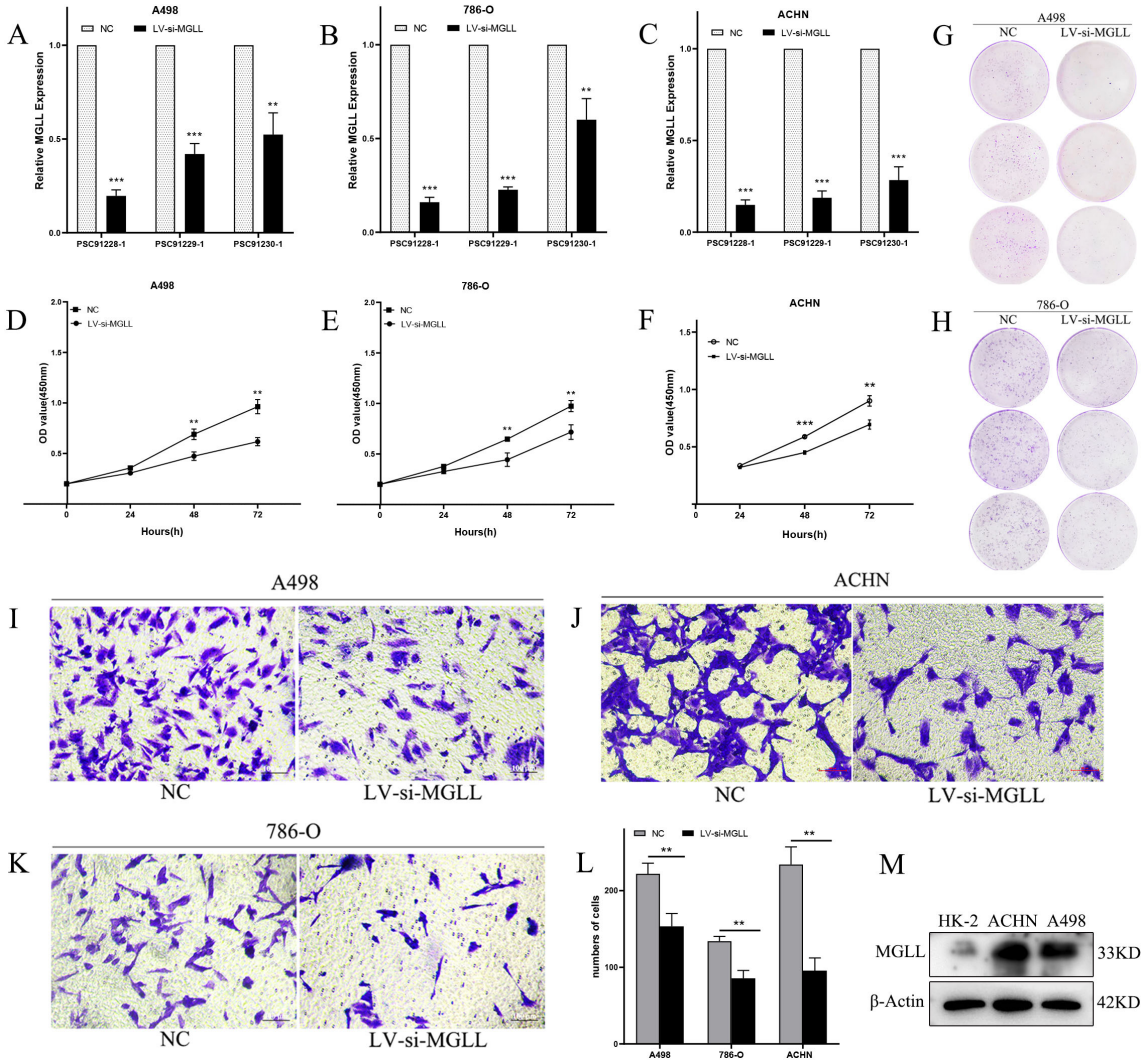
Knockdown of MGLL expression inhibits the proliferation, colony formation, and migration abilities of ccRCC cells. **(A-C)** The knockdown efficiency of three LV-RNAi sequences on MGLL gene expression in A498, 786-O and ACHN cell lines was detected using qRT-PCR. **(D-F)** CCK-8 assay to determine the proliferation levels of A498, 786-O and ACHN cells. **(G, H)** Determination of colony formation experiments in A498 and 786-O cells. **(I-L)** Transwell assay to determine the migration ability of A498, ACHN and 786-O cells (Scale bar, 100 μm). **(M)** Immunoblot assay of MGLL protein expression in normal renal tubular epithelial cells (HK-2) and ccRCC cell lines (ACHN and A498). **p < 0.01; ***p < 0.001.

To elucidate the functional role of MGLL, we performed lentivirus-mediated knockdown experiments and assessed its biological implications. qPCR analysis of three LV-RNAi constructs in ccRCC cell lines (A498, 786-O, and ACHN) identified the PSC91228–1 sequence as the most effective in suppressing MGLL expression, achieving an inhibition rate of 80%-90% (**Figures 8A-C**). This construct was subsequently utilized for further functional assays. CCK-8 assays demonstrated that MGLL knockdown significantly reduced the proliferative capacity of A498, 786-O, and ACHN cells (**Figures 8D-F**). Consistent with these findings, plate colony formation assays revealed that MGLL silencing markedly impaired the colony-forming ability of A498 and 786-O cells (**Figures 8G, H**). Moreover, Transwell migration assays indicated that MGLL knockdown effectively suppressed the migratory potential of A498, 786-O, and ACHN cells (**Figures 8I-L**). To validate these observations, we conducted Western blot analysis in ACHN and A498 cell lines, which exhibited the highest MGLL expression levels based on qPCR results (**Figure 1H**). The Western blot data confirmed that MGLL protein expression was significantly elevated in ACHN and A498 cells compared to normal renal tubular epithelial cells (HK-2) (**Figure 8M**), corroborating our previous findings.

## 4 Discussion

This investigation employed a comprehensive bioinformatics approach, integrating multi-omics data from diverse databases to systematically evaluate MGLL expression in ccRCC. The utilization of extensive datasets was designed to enhance the robustness of our findings, which were subsequently validated through experimental approaches. The primary goal of this research is to clarify MGLL’s role in ccRCC and its potential as a predictive biomarker. We specifically focus on MGLL’s association with clinical outcomes, protein interactions, immune cell infiltration, and m7G methylation, as these factors are implicated in cancer biology and may influence therapeutic responses (33). Through the integration of clinical data, functional clustering analysis, and gene correlation studies, we aimed to establish a comprehensive understanding of MGLL’s impact on tumor behavior and patient survival outcomes, potentially informing novel therapeutic strategies targeting this molecule.

Building upon the established characteristics of lipid metabolism reprogramming in ccRCC, we identified MGLL from lipid storage-associated genes through RNA sequencing analysis, revealing its potential biological properties. Multi-database analysis and experimental validation consistently demonstrated elevated MGLL expression in ccRCC compared to normal renal tissues. Pan-cancer analysis revealed significant expression variations of MGLL across multiple tumor types, suggesting its potential involvement in core regulatory mechanisms of common oncogenic pathways. The functional significance of MGLL has been documented in various malignancies, including endometrial adenocarcinoma (34), lung adenocarcinoma (35), non small cell lung cancer (36), melanoma (37–39), hepatocellular carcinoma (40), and colon cancer (41). Notably, MGLL exhibits both upregulation and downregulation patterns across different tumor types, potentially reflecting tissue-specific adaptations, metabolic or microenvironmental influences, gene functional pleiotropy, epigenetic modifications, or transcriptional regulatory mechanisms (42). Emerging evidence demonstrates that ABX-1431, a novel MGLL inhibitor, effectively reverses progesterone resistance and enhances progesterone sensitivity in endometrial adenocarcinoma (34). Concurrently, recent investigations have identified acetyl-11-keto-β-boswellic acid (AKBA) as another potent MGLL inhibitor, demonstrating therapeutic potential in addressing non-alcoholic fatty liver disease and its associated metabolic complications, including weight gain and insulin resistance (43). These findings suggest that pharmacological modulation of MGLL represents a promising therapeutic strategy, with anticipated advancements in its clinical applications.

Clustering analysis of genes co-expressed with MGLL indicates that the associated biological processes and pathways predominantly involve protein processing in the ER, ERs, macroautophagy, and proteasomal protein catabolism. MGLL-docosahexaenoic acid has been shown to induce ERs via lipid peroxidation, subsequently triggering apoptosis and autophagy in breast cancer cells and xenograft tumors (44). Furthermore, MGLL modulates the E3 ligase activity of target proteins through direct protein interactions, thereby promoting their autoubiquitination and subsequent degradation (42). The ER plays a pivotal role in regulating calcium homeostasis, lipid metabolism, protein synthesis, and post-translational modifications (45). The interplay between ERs and lipid metabolism significantly exacerbates the aggressive phenotype of ccRCC. ERs influences lipid metabolism by altering the expression levels of key enzymes involved in lipid synthesis and metabolic pathways. Conversely, lipids can impact ER functionality and the unfolded protein response through mechanisms such as modifying ER membrane composition and modulating signaling pathways that mediate the ERs response (46). The processes of lipid anabolism, catabolism, and distribution are partially regulated by the ER. Perturbations in lipid metabolism and ERs can disrupt various cellular functions, thereby contributing to the progression of multiple diseases (47). Additionally, the identification of a PPI network involving MGLL provides a foundation for elucidating its biological role in ccRCC. By characterizing co-expressed genes and their associated functional pathways, we can decipher the intricate regulatory networks in which MGLL participates, potentially uncovering novel therapeutic targets (35, 48).

The tumor microenvironment (TME) and tumor-infiltrating immune cells are pivotal in modulating cancer progression (49). Reports have indicated that cuproptosis characteristics serve as immunotherapy biomarkers for ccRCC (50). Tumor-associated neutrophils are known to influence tumor progression (51). In RCC, dirty necrosis is characterized by neutrophil infiltration and cellular debris, which is associated with elevated levels of neutrophil extracellular traps (NETs) (52), NETs may shield cancer cells from T lymphocyte or NK cell-mediated cytotoxicity and facilitate tumor cell capture, promoting distant metastasis (53, 54). MGLL-derived lipid metabolites may regulate the release of myeloid cell cytokines, thereby shaping the local immune microenvironment. In the field of cancer immunotherapy translation, significant progress has been achieved. Circulating biomarkers in blood-based liquid biopsies can help identify which melanoma patients are most likely to benefit from immune checkpoint inhibitor therapy (55). Novel strategies combining genetic engineering and metabolic reprogramming to metabolically adapt chimeric antigen receptor (CAR) T-cells may provide new insights into addressing immune evasion and metabolic suppression in solid tumors (56). Tailored combination checkpoint approaches for different TME can further enhance the efficacy of immunotherapy (57). Additionally, small extracellular vesicles (sEVs) released by tumor cells can carry various bioactive components such as miRNAs, lncRNAs, proteins, and lipids, which can modulate macrophage polarization states. Effectively utilizing sEVs as therapeutic carriers to intervene in the TME may emerge as a potent anti-tumor immune strategy (58).

This study investigated the association between m7G RNA methylation modification genes and MGLL expression in ccRCC. Analysis of the TCGA ccRCC dataset demonstrated significant differential expression patterns among most methylation genes correlated with MGLL, despite limited existing literature specifically addressing their roles in RCC. To validate these findings, we integrated differentially expressed genes from the PPI network with m7G methylation genes, subsequently correlating these data with clinical parameters for prognostic and diagnostic evaluation. Further validation was conducted using the GEO dataset, with final confirmation achieved through qPCR experiments. Our results identified several co-expressed and functionally associated genes with MGLL, including ACLY, CALM3, NSUN2, PKM, NUDT16, and NUDT4, which exhibit potential as valuable prognostic and diagnostic biomarkers for ccRCC. Notably, NUDT16, a member of the nudix hydrolase family, plays a critical role in mRNA metabolism through its decapping activity. As an essential component of the decapping enzyme complex, NUDT16 facilitates mRNA degradation by catalyzing the hydrolysis of the m7GpppN cap structure, thereby selectively eliminating aberrant transcripts and maintaining mRNA quality control mechanisms (59). In contrast to conventional proteomics and transcriptomics methodologies that frequently depend on manual inspection by trained personnel, resulting in labor-intensive and non-standardized procedures, RNA-seq technology offers comprehensive datasets for biomarker identification, yet numerous potential biomarkers remain undetected. This study presents a novel application of generative adversarial networks (GANs) integrated with bioinformatics approaches and diverse computational algorithms to efficiently process and interpret transcriptomic data. This framework not only mitigates the scarcity of gene expression data associated with phenotypic state transitions but also establishes a robust platform for the identification of clinically relevant biomarkers (60).

Our study has systematically investigated the clinical implications of a prognostic model integrating MGLL and its associated gene network. The risk stratification score derived from this model demonstrates predictive accuracy for 5-year overall survival in patients. The prognostic panel, developed through LASSO regression and network-based methodologies, offers substantial clinical research utility. Previous investigations have employed machine learning algorithms for molecular subtyping and prognostic model construction in breast cancer, identifying CRTAM, CLEC2D, and KLRB1 as pivotal hub genes associated with exhausted CD8+ T cell phenotypes (61). Furthermore, survival models based on the MAPK signaling pathway and IGFBPs have delineated core molecular signatures, providing potential therapeutic targets for glioma diagnosis and personalized treatment (62, 63). In contrast to these bioinformatics-driven approaches, our current research focuses on MGLL, establishing the prognostic significance of six signature genes through comprehensive prognostic modeling and diagnostic ROC curve analysis, subsequently validated through experimental approaches and multi-cohort data verification. This integrated analytical framework, combining computational modeling with experimental validation, provides a more rigorous and objective assessment of molecular markers’ functional roles in tumor biology.

This study has the following limitations. First, the analysis of immune infiltration and m7G methylation was conducted through correlation analysis, and further direct experimental evidence is required to confirm the regulatory role of MGLL in the relevant gene network. Future prospective validation will significantly enhance its reliability. Alternatively, immunohistochemistry or NanoString assays in independent patient cohorts could be added to confirm the *in situ* association of cell types. Emerging liquid biopsy technology represents a minimally invasive diagnostic approach with significant potential for real-time patient monitoring and clinical translation. This technique enables the analysis of genetic alterations and drug-resistant mutations through the detection of circulating tumor RNA and circulating tumor cells (64). The integration of liquid biopsy with molecular barcoding technology has demonstrated enhanced detection rates and improved sensitivity in the genomic analysis of pancreatobiliary malignancies (65). Furthermore, specific DNA methylation biomarkers, including epigenetic modifications in APC, RASSFI, and FOXA1, are emerging as promising candidates for a gene panel that could facilitate the early detection of breast cancer via non-invasive liquid biopsy methodologies (66). Second, the experimental results are limited to *in vitro* cell line models. Future *in vivo* investigations of MGLL knockdown or small molecule inhibitors in xenotransplantation will further explore its therapeutic potential. We also note that patient-derived xenograft (PDX) models preserve patient-specific histological features and molecular characteristics, which makes them ideal models for evaluating tumor behavior and drug response (67). Future studies may also consider constructing PDX models of tumors related to obesity (68, 69). These studies would investigate the direct effects of MGLL inhibitors on tumor growth. Third, more clinical samples and cohort data need to be included to reduce heterogeneity and enhance the robustness of the research findings. In addition, patient stratification should be implemented to improve the accuracy and clinical applicability of the results. Future analyses need to be conducted through further mechanistic studies.

In conclusion, our findings demonstrate the critical involvement of MGLL in ccRCC pathogenesis and its potential utility as a diagnostic and prognostic biomarker. The complex interplay between MGLL expression, clinical characteristics, immune cell infiltration, and molecular interactions reveals a multifaceted biological network that necessitates further comprehensive investigation.

## Data Availability

The original contributions presented in the study are included in the article/**Supplementary Material**. Further inquiries can be directed to the corresponding authors.
