# Supplementary material for: Comprehensive analysis of MGLL as a novel diagnostic and prognostic biomarker for clear cell renal cell carcinoma
Source: Front Oncol. 2025 Sep 9;15:1594540. doi: 10.3389/fonc.2025.1594540 (PMC12454099; doi:10.3389/fonc.2025.1594540)
Supplement: Supplementary file 5 [file Table1.docx]

Supplementary Materials

# Supplementary Tables

**Table S1** The primer sequences used are listed.

Sequence (5’ to 3’):

MGLL-F: CGAAGGGGAGAGGATGGTAGT

MGLL-R: GGAAGCCCAGGGTAGTCTTT

ACLY-F: ATCATCTCTCGGACCACGGA

ACLY-R: GCAGACGATGGGCTTAGTGA

AGO2-F: TAGACCCGACTTTGGGACCT

AGO2-R: GGGCACTTCTCTGGCTTGAT

CALM3-F: GGCACTATCACCACCAAGGA

CALM3-R: AAGTCAATGGTCCCGTTCCC

CYFIP1-F: CAAGAATCAGGGAGCCACGA

CYFIP1-R: TCTATTGATCTGCCGAGGAGC

EIF4E3-F: CTGTTAGCAACCATCGGGGA

EIF4E3-R: GGTCCCGAACACTGACACTAA

GEMIN5-F: AGGCGTGAAGTCCAAGGTTAC

GEMIN5-R: GGAGGCTTGTTGGAGTAGGTG

LSM1-F: ACAAAGGGTGGAACAGCAGAC

LSM1-R: CAGTGACAGCCCCTACTCTTC

NSUN2-F: TGGTTTACAGACTGGGACGC

NSUN2-R: GCTTTTCTGGGTCCTTCGGA

NUDT16-F: ACACGCAGGACAGAAGCCTA

NUDT16-R: AGACGCTTGGCATAGAAGTGG

NUDT4-F: TTTGAGCAGAACCAAGACCGA

NUDT4-R: CGGAAGGGAAGGGACTGTAGA

PKM-F: AATCACGCTGGATAACGCCT

PKM-R: TCGGCACCTTTCTGCTTCAC

SEC61A1-F: ATTTCCAGGGCTTCCGAGTG

SEC61A1-R: CGAGCTGAGAGCATTTGGGA

UMPS-F: GAGGGCTTGAAGGTCACTGAT

UMPS-R: TTCGCTGCCACAAAGACATT

GAPDH-F: GCACCGTCAAGGCTGAGAAC

GAPDH-R: TGGTGAAGACGCCAGTGGA

β-actin -F: CATGTACGTTGCTATCCAGGC

β-actin -R: CTCCTTAATGTCACGCACGAT

**Table S2** Detailed information on 751 differentially expressed genes.

**Table S3** Detailed information on 2008 co-expressed genes (|correlation spearman| > 0.4 and *p.adj* < 0.05).

**Table S4** The detailed results of the GO analysis and KEGG pathway analysis.

**Table S5** Correlation analysis of MGLL expression levels and m7G methylation genes in ccRCC.

# Supplementary Figure


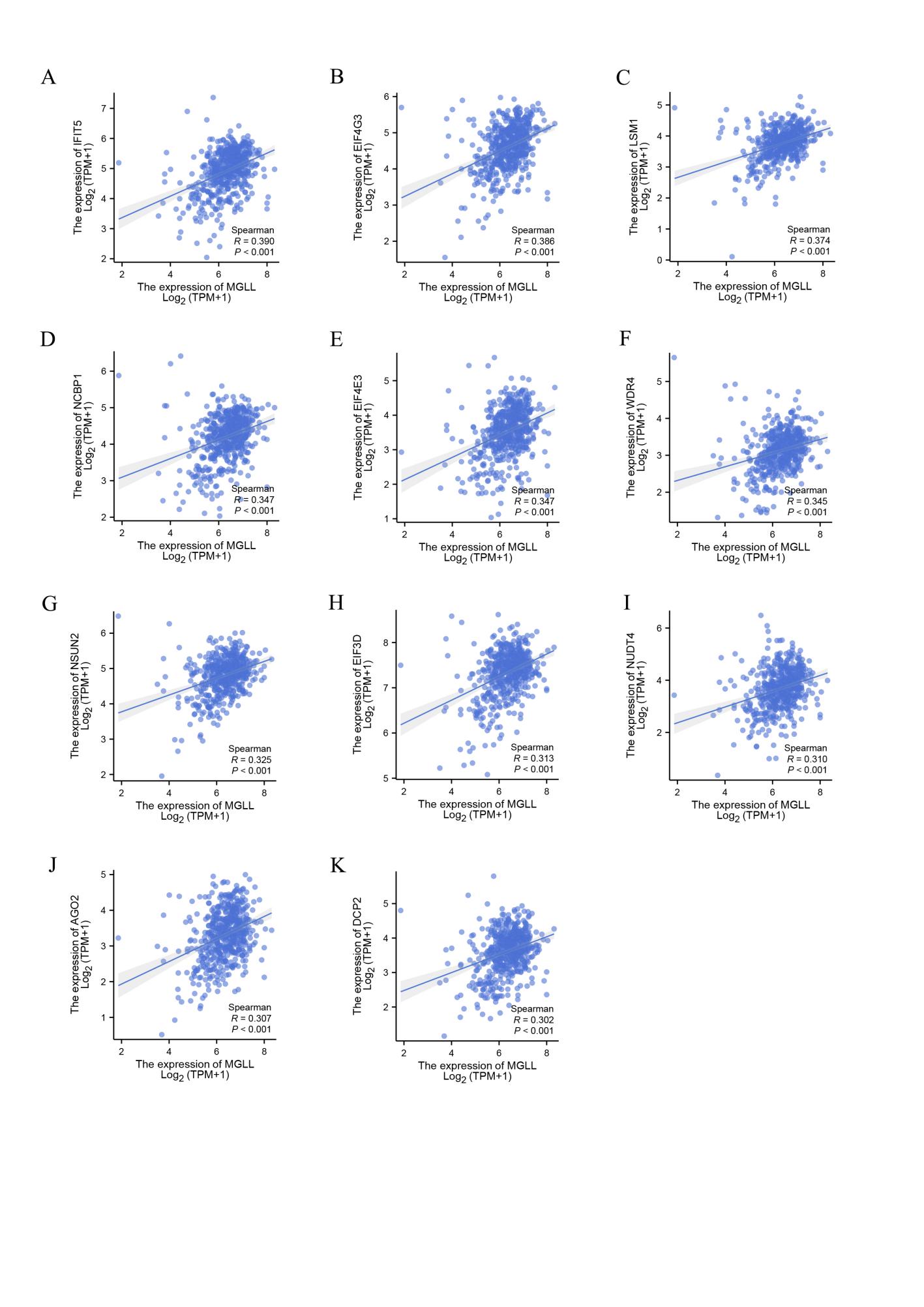


**Supplementary Figure S1.** The scatter plot of genes with a correlation coefficient r > 0.3 related to MGLL expression. (*p* < 0.001)
